# Highly Sensitive Hybridization Chain Reaction-Based miRNA Detection Technology Using Diffusivity Analysis of Fluorescent Probe-Modified miRNA Particles

**DOI:** 10.3390/s26020713

**Published:** 2026-01-21

**Authors:** Momoka Nakai, Yui Watanabe, Maho Koda, Chisato Sakamoto, Tatsuhito Hasegawa, Han-Sheng Chuang, Hiroaki Sakamoto

**Affiliations:** 1 Department of Frontier Fiber and Technology and Science, Graduate School of Engineering, University of Fukui, Fukui 910-8507, Fukui, Japan; 2 Chemistry and Biology, National Institute of Technology, Fukui College, Sabae 916-0064, Fukui, Japan; 3 Fundamental Engineering for Knowledge-Based Society, Graduate School of Engineering, University of Fukui, Fukui 910-8507, Fukui, Japan; t-hase@u-fukui.ac.jp; 4 Department of Biomedical Engineering, National Cheng Kung University, Tainan 701401, Taiwan; oswaldchuang@mail.ncku.edu.tw

**Keywords:** MicroRNA, probe-modified particle, image analysis, hybridization chain reaction, photo-crosslinkable artificial nucleic acid

## Abstract

**Highlights:**

**What are the main findings?**

- This research aims to develop a novel and highly sensitive detection method for microRNA (miRNA) in complex biological samples like serum.
- The study addresses the challenge of achieving both high selectivity and sensitivity by combining image analysis of particle diffusivity with ad-vanced signal amplification techniques.

**What are the implications of the main findings?**

- We successfully developed a sensor that monitors the diffusivity change of fluorescent probe-modified particles after Hybridization Chain Reaction (HCR) amplification.
- The key innovation is the use of a photo-crosslinkable artificial nucleic acid probe, which forms a covalent bond with the target miR-21 to ensure robust capture. This method demonstrated high selectivity and a remarkable detection limit as low as 1 fM in serum.

**Abstract:**

MicroRNAs (miRNAs) are promising biomarkers for the early detection of various diseases, particularly cancer, driving active development of highly sensitive and selective detection technologies. This study aims to establish a novel miRNA detection technique that utilizes image analysis to track the Brownian motion (diffusivity) of fluorescent probe-modified miRNA particles. This method identifies the presence and concentration of miRNAs by exploiting the change in particle size upon hybridization with the target. Furthermore, the use of a probe modified with a photo-crosslinkable artificial nucleic acid (CNV-D) enables the covalent capture of the target miRNA, ensuring high selectivity in biological samples even under stringent washing conditions. By integrating Hybridization Chain Reaction (HCR), the complex size is significantly amplified, dramatically enhancing the detection sensitivity. Consequently, we successfully demonstrated the highly sensitive and specific detection of the cancer biomarker miR-21 in serum, achieving an exceptionally low limit of detection (LOD) of 1 fM. This technology holds great potential to contribute to the early diagnosis of cancer.

## 1. Introduction

Early diagnosis of cancer is important. Imaging techniques, such as radiography, magnetic resonance imaging, computed tomography, and positron emission tomography, are commonly used to diagnose cancer [1,2,3,4]. Another method used for diagnosing cancer is biopsy, which involves removing a portion of the lesion and closely examining it under a microscope. However, the above-mentioned techniques are invasive and depend on the phenotypic characteristics of the tumor, making early detection of cancer next to impossible. Recently, microRNA (miRNA) detection has attracted considerable attention as an early cancer detection technique [5,6]. miRNAs are non-coding RNA molecules that control gene expression at the post-transcription level by targeting messenger RNAs and are approximately 22 nucleotides long. miRNAs are known as cancer diagnostic biomarkers because the changes in their expression levels can indicate the presence of cancer [7,8]. DNA microarrays and quantitative reverse transcription polymerase chain reaction (RT-qPCR) are currently used to detect miRNAs [9,10,11]. DNA microarrays can detect target miRNAs by labeling them with fluorescent dyes, which are then hybridized to complementary DNA probes on the array, allowing for detection and quantification of the miRNAs. DNA microarrays offer the advantage of performing a comprehensive analysis on multiple types of miRNAs. RT-qPCR is a highly sensitive target amplification method for detecting miRNAs, even at low concentrations. However, miRNAs typically contain a small number of bases (nucleotides); thus, designing primers for quantitative RT-qPCR to detect microRNAs can be challenging. Surface-enhanced Raman scattering (SERS) and electrochemical detection have been utilized as techniques for detecting miRNAs [12]. SERS is a rapid and non-destructive technique, which has high sensitivity and specificity; however, SERS is expensive and not fast or easy to use. Electrochemical detection is easy to miniaturize and involves relatively simple, low-cost equipment; however, fabricating an electrochemical biosensor to simultaneously detect multiple cancer analytes is difficult [13,14,15].

This study aims to establish a miRNA detection technique based on image analysis. Image analysis can be performed in a short time and does not require complicated or expensive machinery. Chuang et al. developed a method to quantify bacterial growth by combining an optical diffusion assay with a particle-based immunological assay [16,17]. Particles in a solution undergo translational Brownian motion, which can be expressed by the Stokes–Einstein equation. The particle size and translational diffusivity are inversely proportional when the ambient temperature and fluid viscosity are properly controlled. In other words, particles with smaller diameters exhibit greater diffusivity, whereas those with larger diameters exhibit smaller diffusivity. Diffusion decreases as particle size increases when biomolecules form complexes with fluorescent particles in antigen–antibody reactions. In our previous study, we constructed a sensing system based on the method developed [18,19,20,21] for nucleic acid detection and successfully detected genomic DNA (approximately 2.84 Mbp) of methicillin-resistant Staphylococcus aureus (MRSA) [22]. The designed oligonucleotide probe was modified using gold nanoparticles and fluorescent particles to form a complex with the target nucleic acid. The modified probe enabled the detection of nucleic acids by monitoring the change in the particle size of the complex based on the concentration of MRSA and measuring the change in diffusivity of MRSA. Notably, the diffusion motion-based detection method has the advantage of being based on Brownian motion, which is a self-driving force that remains unaffected by power failure and simplifies sample pre-processing. Previously, we reported a technique for collecting and detecting miRNAs using magnetic particles modified with probes that have sequences complementary to the target miRNAs [23]. We demonstrated a highly efficient miRNA detection technique by introducing photo-crosslinkable artificial nucleic acids into a probe to complement the target nucleic acids [24]. In this study, we developed a sensor that can identify the presence or absence of miRNAs using image analysis. The sensor was developed by integrating the existing technology based on magnetic particles modified using probes with one that detects the Brownian motion of miRNA particles using image analysis. To date, image analysis technology has been successfully demonstrated to detect the MRSA genome and coronavirus RNA [25]. The technology proposed in this study is based on the decrease in diffusivity of miRNA particles owing to the increase in particle size caused by the formation of complexes between the target nucleic acids and probe-modified particles.

**Figure 1:**
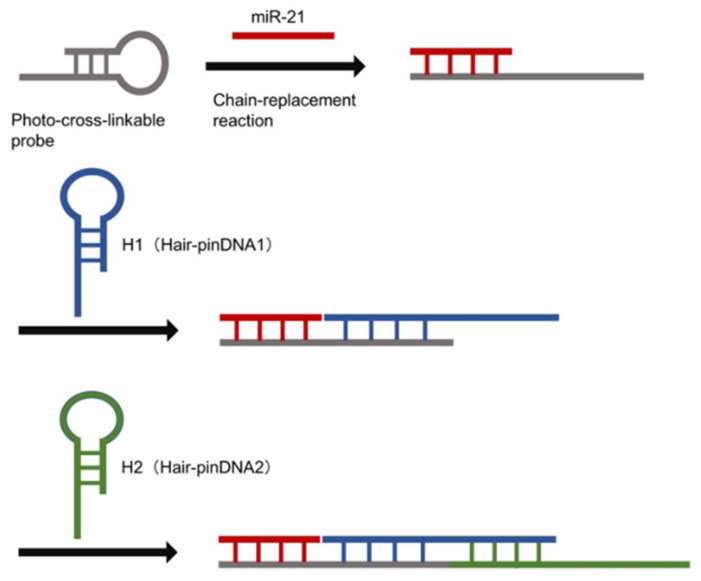
Schematic of the hybridization chain reaction involving two metastable DNA hairpin structures.

**Figure 2:**
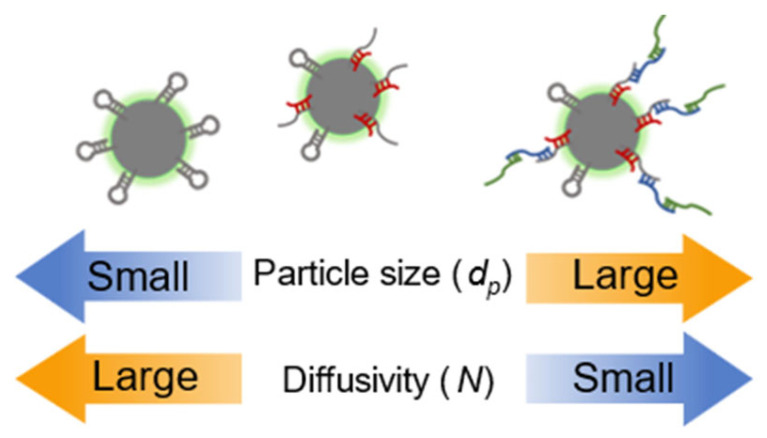
Variation in diffusivity with particle size using HCR.

The miRNAs targeted in this study are short chains; thus, the change in particle size is expected to be small. Consequently, the change in diffusion is also expected to be small, making the detection of target miRNAs more difficult. Therefore, we focused on hybridization chain reaction (HCR) as a nucleic acid amplification technique for particle size expansion after target recovery. In HCR, single-stranded nucleic acids recognize the terminal single-stranded portion or toehold region of hairpin nucleic acids sequentially and initiate a complex substitution reaction [26,27,28]. The chain displacement and complex substitution reactions occur between the single-stranded portions of DNA hairpin 1 (H1) and hairpin 2 (H2). The reactions result in opening the hairpin structures of H1 and H2 (Figure 1). Subsequently, the spontaneous hybridization of H1 and H2 occurs via a similar chain reaction. The HCR results in a long hybridization product with a single-stranded nucleic acid as the starting sequence. The HCR also increases the size of the complex, increasing the sensitivity of image analysis. To fabricate the probes for collecting and detecting miRNAs, photo-crosslinkable artificial nucleic acids containing 3-cyanocarbazole-modified nucleotides (CNV-D) were introduced into the probes. Oligodeoxynucleotides (ODN) containing CNV-D were introduced because they can be crosslinked with pyrimidine bases that are present between the two nucleic acid strands of miRNAs upon irradiation using 366 nm light for 1 s. The carbon-to-carbon double bond of CNV-D and the C5 = C6 double bond of the pyrimidine base of the target nucleic acid form a covalent bond via [2 + 2] cycloaddition reaction upon photoirradiation [29,30,31]. Formation of hydrogen bonds is also possible via hybridization of DNA probes with photo-crosslinkable artificial nucleic acids and target miRNAs, resulting in the formation of a covalent bond between photo-crosslinkable artificial nucleic acids and target miRNAs by photoirradiation; this, in turn, facilitates the breaking of the existing hydrogen bonds in the target miRNAs, removing foreign substances while still capturing the target miRNAs and increasing the specificity of the HCR. By combining artificial probe technology and image analysis, this study proposed a rapid, simple, and highly sensitive miRNA detection method using HCR. The proposed technology is based on image analysis to determine the Brownian motion of fluorescent probe-modified miRNA particles (Figure 2). In this study, miR-21, a small non-coding RNA molecule whose expression increases in various cancer cells, was used as the target miRNA.

Compared with previously reported miRNA probes, the present sensing system offers several innovations. First, the photo-crosslinkable artificial nucleic acid (CNV-D) enables covalent capture of the target miRNA after hybridization. This irreversible binding allows stringent washing while maintaining the captured target, greatly reducing nonspecific adsorption, particularly in serum. Second, our method analyzes the diffusivity of probe-modified particles rather than relying solely on fluorescence intensity. Because Brownian motion provides a self-driven signal that does not require complex instrumentation, the detection principle remains simple and robust. Third, the hybridization chain reaction (HCR) amplifies the hydrodynamic size of the probe–miRNA complex, resulting in a substantial improvement in detection sensitivity without enzymatic amplification. Through the synergistic combination of photo-crosslinking, diffusivity-based sensing, and HCR amplification, the proposed probe achieves highly sensitive and selective miRNA detection while maintaining operational simplicity.

## 2. Materials and Methods

### 2.1. Preparation of Photo-Crosslinkable Probe-Modified Fluorescent Particles

To prepare a fluorescent particle solution, fluorescent streptavidin-coated polymer microspheres (Diameter: 0.2 µm; excitation wavelength: 480 nm, fluorescence wavelength: 520 nm; Bangs Laboratories, State of Indiana, US) were suspended in a binding and washing buffer [1×]. Next, 10 µL of the prepared fluorescent particle solution was taken in a container and 6.06 µL of 10 µM photo-crosslinkable probe was added to it. Subsequently, the volume of the mixed solution was brought to 10 µL using the binding and washing buffer [1×]. To form a hairpin structure, the biotin-modified photo-crosslinkable probes were incubated at 95 °C for 5 min, at room temperature for 15 min, and on ice for 5 min. The probe was immobilized on the particle surface via a biotin-avidin interaction by agitation at room temperature for 15 min. The unmodified probes were then removed using 10 µL binding and washing buffer [1×]. Finally, the obtained solution was dissolved in 10 µL of binding and washing buffer [1×] to obtain a photo-crosslinkable probe-modified fluorescent particle solution.

### 2.2. Sample Preparation

To dissociate DNA from a higher-order structure, 1 µM photo-crosslinkable probe, 10 µM H1, and 10 µM H2 were heated at 95 °C for 5 min. Subsequently, the DNA hairpin structures, H1 and H2, were incubated at room temperature for 15 min and on ice for 5 min. Next, 5 M NaCl, 1 M MgCl_2_, 1 M Tris-HCl buffer (pH 7.7), and 10 (*v*/*v*) Tween 20 were diluted to final concentrations of 15 mM, 25 mM, 1 mM, and 0.001 (*v*/*v*), respectively, in water. The diluted solutions were mixed to prepare the HCR buffer. In the HCR buffer, probe-modified fluorescent particles and miR-21 were hybridized at 37 °C for 60 min. Irradiation was performed at 366 nm for 1 min to form covalent bonds between the probe and miR-21. A 6 M urea solution was used to cleave the hydrogen bonds from hybridized miR-21 to remove foreign substances and increase non-specific adsorption. Next, H1 and H2 were added to the HCR buffer containing the hybridized miR-21, and the buffer was incubated at 37 °C for 80 min to allow the HCR to proceed. Finally, a hybridized complex solution was obtained. The movement of the hybridized complex miR-21 particles was tracked over time by recording movies using fluorescence microscopy, and the recorded movies were divided into images using the Image J software (Version 1.54 g 18). More details regarding the imaging are provided in Section 2.3. The diffusivity of the hybridized complex miR-21 particles was calculated by determining the change in position of the particle after 0.066 s using a dedicated program.

### 2.3. Detection by Image Analysis

**Figure 3:**
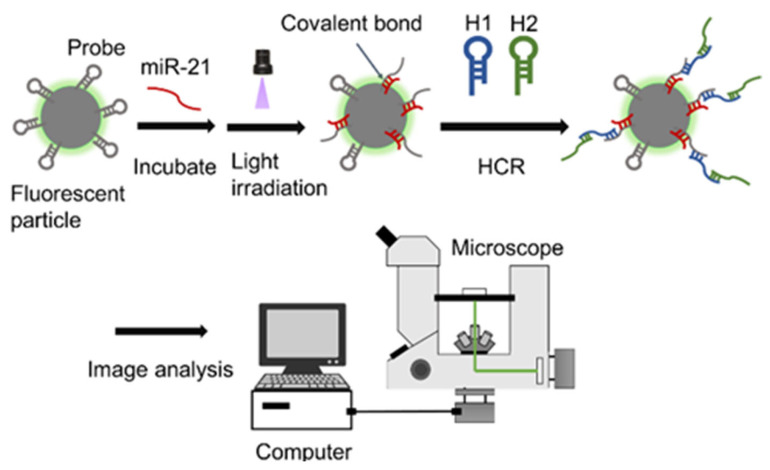
Schematic depicting the method for detecting miR-21 using image analysis.

A layer of colored kraft and cellophane tapes was applied to a glass slide. Next, 1.5 µL of the hybridized complex solution (described in Section 2.2) was poured on the glass slide and covered with a cover glass to form a thin film measuring ~120 µm. A fluorescence microscope (CKX41, Orympus, Tokyo, Japan) was used to record movies at 15 fps with a resolution of 1824 × 1216 pixels for 20 s using a 40× objective and 10× eyepiece while irradiating the thin film with an excitation light of approximately 470–500 nm (Figure 3). Six measurements were performed for each sample. Using ImageJ, the recorded movies, each 20 s long, were divided into images every 66 ms. The obtained images were analyzed using a dedicated program.

The mean squared displacement is defined as the average of the squares of the distance traveled by the particles from the starting point. Diffusivity is defined as the slope of the horizontal axis as the elapsed time and the vertical axis as the mean squared displacement. The detection algorithm has been previously reported. The relative diffusivity was defined as the percentage ratio of the particle diffusivity in the presence of the target miRNA to that measured in the absence of the target, which was normalized as 100%. The error bars shown represent the standard deviation of six independent measurements (*n* = 6). The limit of detection (LOD) was calculated as the mean relative diffusivity of the blank minus three times its standard deviation (LOD = μ_blank − 3σ_blank), following established analytical chemistry conventions.

## 3. Results

### 3.1. Confirmation of HCR by Agarose Electrophoresis

**Figure 4:**
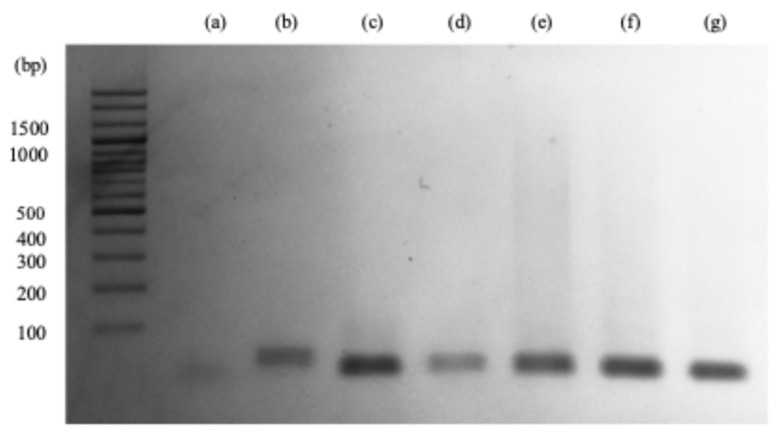
Smear on agarose gel from: (**a**) 1 µM miR-21, (**b**) 1 µM photo-cross-linkable probe, (**c**) 1 µM H1, (**d**) 1 µM H2, (**e**) miR-21/photo-cross-linkable probe/H1/H2, (**f**) photo-cross-linkable probe/H1/H2; (**g**) H1/H2.

Agarose gel electrophoresis was performed to confirm the progression of HCR (Figure 4). Sample (e), the hybridized complex miR-21 (described in Section 2.2), exhibits the longest and darkest smear. The miR-21, photo-crosslinkable probe H1, and H2 are 22, 48, and 50 nt long, respectively. The smear extends to approximately 1500 bp in the presence of miR-21. The presence of miR-21, based on the smear analysis, suggests that a photo-crosslinkable probe-modified miR-21 complex is formed, and HCR progresses via the successive opening of two nucleic acid hairpins, H1 and H2. The maximum number of hybridization products formed as a consequence of the HCR is ~30. The H1/H2 complexes after reacting with the miR-21 probe exhibit a smear, which extends to approximately 1500 bp.

### 3.2. Sensor Performance Evaluation

**Figure 5:**
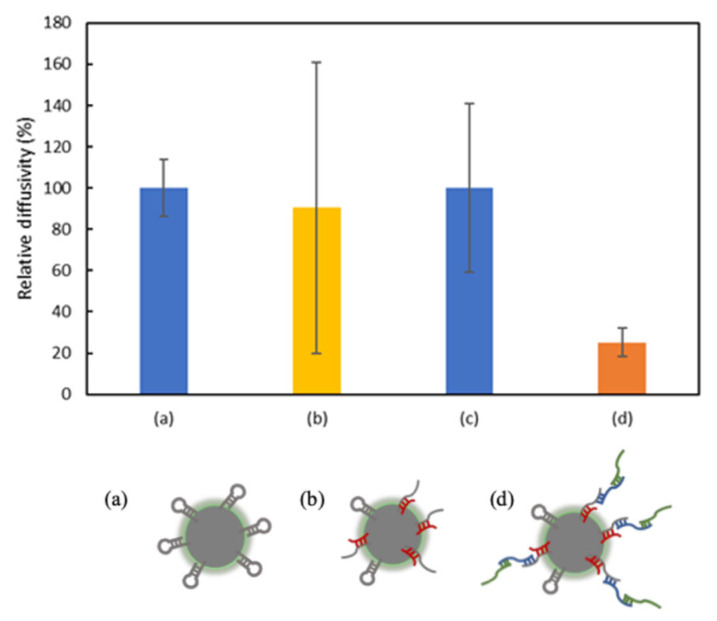
Detection of miR-21 and evaluation of the effectiveness of HCR. Graph showing relative diffusivities of (**a**) without miRNA and HCR, (**b**) with miR-21 and without HCR, (**c**) without miRNA and with HCR, and (**d**) with miR-21 and HCR. (*n* = 6).

**Figure 6:**
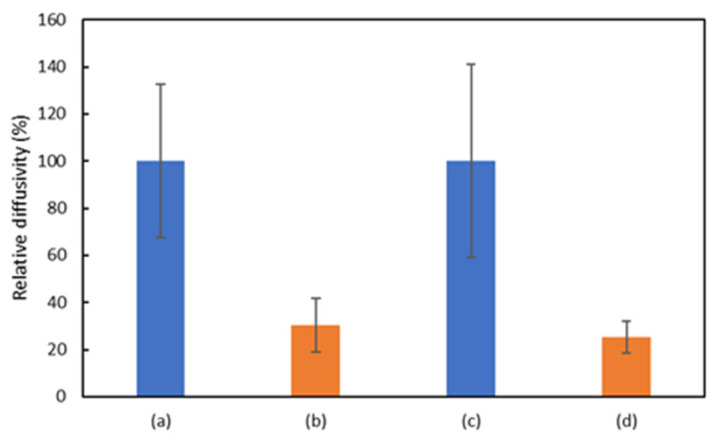
Evaluation of the selectivity of the sensor in serum. Graph showing the relative diffusivities of (**a**) without miR-21 in HCR buffer, (**b**) with miR-21 in HCR buffer, (**c**) without miR-21 in serum, and (**d**) with miR-21 in serum. (*n* = 6).

miR-21 detection performance of the photo-crosslinkable probe-modified fluorescent particles via HCR was tested using image analysis (Figure 5). The result shows that the diffusivity of sample (b) in the presence of miR-21 is lower than that of sample (a) in the absence of miR-21. The lower diffusivity of sample (a) than sample (b) can be attributed to the formation of a photo-crosslinkable probe-modified miR-21 complex in sample (b), which expands the particle size. Therefore, miR-21 can be detected using image analysis by monitoring the change in diffusivity using the prepared sensor. The effectiveness of HCR was evaluated by comparing the diffusivities of samples prepared with and without the HCR. As compared to the diffusivity of sample (a), sample (d), which was allowed to undergo HCR in the presence of miR-21, showed a higher decrease in diffusivity than sample (c), which was allowed to undergo HCR in the absence of miR-21. As compared to the diffusivity of sample (a), the extent of decrease in diffusivity of sample (b) was greater than that of sample (c), suggesting that HCR further increases the particle size and results in a significant decrease in diffusivity. Therefore, the diffusivities of miRNAs reduce when the proposed method utilizing image analysis is employed for detection. The results presented in Figure 5 show that the HCR increases the sensitivity of miR-21 detection performed using image analysis. The larger error observed in Figure 5b originates from the heterogeneous formation of the probe–miRNA complex prior to HCR amplification, which is minimized after HCR, as shown in Figure 5d. The relatively large standard deviations observed in Figure 5b and Figure 6 are attributed to heterogeneity in the formation of the probe–miRNA complexes before HCR amplification. In this stage, incomplete hybridization and uneven photo-crosslinking can cause variations in particle size and diffusivity. After HCR amplification, the polymerized products stabilize the population, resulting in reduced variance as seen in Figure 5d. Additionally, small fluctuations in temperature and viscosity during image acquisition may contribute to the observed measurement error. This demonstrates that HCR contributes not only to signal amplification but also to signal stabilization.

### 3.3. Sensor Performance in Serum

Serum samples containing various proteins and nucleic acids were used to evaluate the sensor selectivity. In Figure 6, the concentration of miR-21 used in both buffer and serum experiments was fixed at 1 nM in order to evaluate selectivity under identical conditions. Figure 6 shows that the diffusivity of the sample (d) decreases in the presence of miR-21 in serum, indicating that the proposed sensor can selectively detect miR-21. In Figure 6, as compared to the diffusivity of sample (a), sample (d) shows the same extent of decrease in diffusivity as sample (b), which was allowed to undergo the HCR buffer, indicating that the proposed sensor can detect miR-21 in serum without loss of sensitivity.

**Figure 7:**
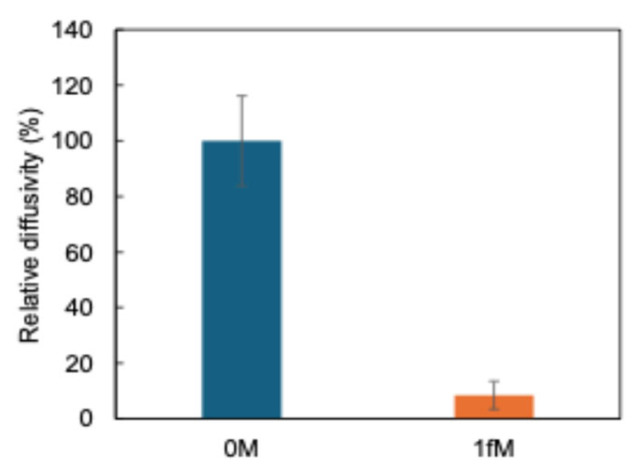
Sensor response in serum: graph showing relative diffusivities of serum components at 0 M and 1 fM miRNA concentrations. (*n* = 6).

Finally, the sensor’s sensitivity was evaluated by varying the concentration of 1 fM miR-21. Figure 7 shows that the diffusivity decreases in all samples containing miR-21 as compared to the sample without miR-21. The observation that the relative diffusivity becomes significantly low even at 1 fM is attributed to the photo-crosslinking process, which enables irreversible capture of the target, followed by HCR-mediated polymer growth that dramatically increases the effective hydrodynamic diameter of the complex. Since Brownian diffusivity scales inversely with particle radius, even a small amount of captured miRNA results in a measurable diffusivity decrease. Therefore, based on the statistically significant difference between the blank and the 1 fM sample, the limit of detection was defined as 1 fM.

## 4. Discussion

We established a novel miRNA detection technology by capturing target miRNAs using photo-crosslinkable probe-modified fluorescent particles and increasing the detection sensitivity using HCR. The proposed technology is based on image analysis to detect the Brownian motion of fluorescent probe-modified miRNA particles. The target miRNA, miR-21, was captured using photo-crosslinkable probes with sequences complementary to miR-21. The introduction of artificial nucleic acid into the photo-crosslinkable probes facilitated the formation of covalent bonds between the probes and miR-21; this, in turn, allowed the removal of foreign substances by breaking the existing hydrogen bonds while efficiently capturing miR-21, even after strong washing. Subsequently, the size of the captured probe-modified miR-21 particles was increased via HCR performed using hairpin DNA H1 and H2. The target RNA, miR-21, was successfully detected using image analysis by calculating the change in diffusivity brought on by the change in particle size. The agarose gel electrophoresis confirmed that miR-21 can be detected using the technology based on a photo-crosslinkable probe and HCR. The HCR-modified diffusivity of the miR-21 particles was significantly lower than that without HCR, indicating that HCR increases the sensitivity of the proposed technology toward miR-21. The results demonstrated that miR-21 can also be detected in serum containing various proteins and nucleic acids using the proposed technology. Additionally, the detection of miR-21 in the serum containing 1 fM of miR-21 demonstrated the high selectivity of the proposed technology. In this study, selectivity was evaluated using miR-21 as a representative cancer-related miRNA. Although the proposed photo-crosslinkable probe provides strong sequence-specific binding, further experiments using non-target miRNAs of similar length and partial sequence overlap, such as miR-141 or miR-155, are necessary to fully assess potential cross-reactivity. This will be investigated in future studies. Compared with RT-qPCR, the proposed method does not require enzymatic amplification or thermal cycling, which significantly simplifies instrumentation and reduces analysis time. For quantitative comparison, RT-qPCR generally achieves a limit of detection (LOD) around 10 aM with high quantification accuracy, while SERS-based assays typically detect miRNAs at approximately 100 aM but require expensive optical components. Electrochemical sensors exhibit LODs in the range of 10–100 fM depending on probe design and surface treatment. In contrast, the present diffusivity-based method achieves an LOD of 1 fM in serum without enzymatic amplification, demonstrating comparable or superior sensitivity with simpler instrumentation. However, RT-qPCR still offers superior quantification accuracy at extremely low concentrations. Compared with SERS-based sensors, the present system avoids expensive optical components and complicated spectral analysis, although SERS provides molecular fingerprinting capabilities. Electrochemical sensors offer compactness and easy integration into portable devices, but often suffer from electrode fouling and multi-step surface modification. In contrast, the present diffusivity-based sensor provides a simple optical readout, high selectivity due to photo-crosslinking, and high sensitivity enabled by HCR. Nevertheless, limitations include the requirement for fluorescence microscopy and the need for careful temperature and viscosity control during diffusivity analysis.

## Data Availability

The original contributions presented in this study are included in the article. Further inquiries can be directed to the corresponding author.
